# Hexosylceramide Species in the Blood Decline in Both COVID-19 and Non-COVID-19 Sepsis

**DOI:** 10.3390/biomedicines14071635

**Published:** 2026-07-20

**Authors:** Vlad Pavel, Patricia Mester, Stephan Schmid, Sabrina Krautbauer, Marcus Höring, Gerhard Liebisch, Martina Müller, Christa Buechler

**Affiliations:** 1 Department of Internal Medicine I, Gastroenterology, Hepatology, Endocrinology, Rheumatology and Infectious Diseases, University Hospital Regensburg, 93053 Regensburg, Germany; vlad.pavel@klinik.uni-regensburg.de (V.P.); patricia.mester@klinik.uni-regensburg.de (P.M.); stephan.schmid@klinik.uni-regensburg.de (S.S.); martina.mueller-schilling@klinik.uni-regensburg.de (M.M.); 2 Institute of Clinical Chemistry and Laboratory Medicine, University Hospital Regensburg, 93053 Regensburg, Germany; sabrina.krautbauer@klinik.uni-regensburg.de (S.K.); marcus.hoering@klinik.uni-regensburg.de (M.H.); gerhard.liebisch@klinik.uni-regensburg.de (G.L.)

**Keywords:** liver cirrhosis, hexosylceramide, sepsis, ventilation

## Abstract

**Background/Objectives:** Hexosylceramides (HexCers) are bioactive lipids whose circulating levels have been associated with severe illness. Blood lipid profiles differ between COVID-19 and non-COVID-19 sepsis and are also affected by liver cirrhosis. To evaluate the impact of SARS-CoV-2 infection and investigate associations with disease severity and underlying liver cirrhosis among circulating HexCer species, we analyzed plasma samples from patients with systemic inflammatory response syndrome (SIRS), sepsis, or septic shock. **Methods:** Plasma levels of five HexCer species were quantified by flow injection analysis tandem mass spectrometry (FIA-MS/MS) in 159 patients with SIRS, sepsis, or septic shock. Among these patients, 24 had COVID-19, and 31 liver cirrhosis. In addition, serum HexCer levels were analyzed in 41 patients with moderate and 61 patients with severe COVID-19. **Results:** Patients with SIRS, sepsis or septic shock exhibited largely comparable plasma HexCer18:1;O2/22:0, 23:0, and 24:0 concentrations, all of which were significantly lower than those observed in healthy controls. These species and in addition HexCer18:1;O2/16:0 and 24:1 levels were modestly lower in patients with septic shock compared to those with SIRS. No significant differences in any of the five HexCer species were observed between patients with COVID-19 and non-COVID-19 septic shock or between ventilated patients with and without COVID-19, indicating that circulating HexCer levels are associated with disease severity rather than SARS-CoV-2 infection. HexCer18:1;O2/24:1 levels were increased in patients with cirrhosis. No significant differences in HexCer levels were observed between survivors and non-survivors. **Conclusions:** In COVID-19 and non-COVID-19 patients circulating levels of HexCer18:1;O2/22:0, HexCer18:1;O2/23:0, and HexCer18:1;O2/24:0 decline early during systemic inflammation, whereas reductions in HexCer18:1;O2/16:0 and 24:1 levels become apparent in septic shock. These findings indicate that alterations in circulating HexCer species reflect sepsis severity rather than being specific to COVID-19.

## 1. Introduction

Sepsis develops when the host immune response to infection becomes dysregulated, resulting in life-threatening organ dysfunction and a substantial burden of morbidity and mortality [1,2,3]. Bioactive lipid mediators govern the magnitude and resolution of inflammation by modulating both pro-inflammatory and anti-inflammatory pathways. This dual involvement substantially influences disease progression and clinical outcomes [4,5,6].

As bioactive sphingolipids, ceramides participate in diverse cellular processes, including metabolic regulation and inflammatory signaling [7,8,9,10]. Previous studies have demonstrated that patients with sepsis exhibit increased circulating levels of ceramide 18:1;O2/16:0, 18:0, 20:0, and 24:1, accompanied by reduced levels of ceramide 18:1;O2/23:0, 24:0, and 26:0 [11,12]. Notably, ceramide profiles did not differ significantly between sepsis patients with and without SARS-CoV-2 infection, suggesting comparable regulation of these lipid species in the blood irrespective of COVID-19 status [12].

Hexosylceramides (HexCers) are generated from ceramides by hexosylceramide synthase and serve as precursors for most of the mammalian glycosphingolipids, such as gangliosides [13]. Compared to ceramides, the function of hexosylceramides have been only marginally studied [13]. These lipids are essential for cell viability, cancer, and inflammatory responses [13].

In the serum of mice infected with SARS-CoV-2, HexCer 14:0, 16:0, 18:1, and 22:0 levels were increased, whereas HexCer 23:0 levels were decreased [14]. However, these alterations were not associated with disease severity, and HexCer levels were comparable between symptomatic and asymptomatic animals [14].

Reduced serum HexCer levels have been reported in patients with COVID-19 compared with healthy controls [15]. These reductions did not normalize during follow-up and remained evident at days 4–6 after diagnosis [15]. Decreased serum HexCer levels were also observed in patients with non-COVID-19 infectious diseases, suggesting that HexCer reduction may reflect a general response to infection rather than a COVID-19–specific effect [15].

Disease severity–dependent differences in individual HexCer species have also been described. Patients with mild COVID-19 exhibited higher levels of HexCer 20:0 and 22:0 compared to those with moderate disease [16]. Moderate cases showed lower levels of HexCer 16:0, 20:0, and 24:0 than severe cases, whereas severe cases had higher HexCer 24:0 levels than critically ill patients [16]. In a second cohort, total HexCer levels in patients with less severe COVID-19 were comparable to those of healthy controls, whereas patients with more severe disease exhibited reduced levels [15]. Conversely, another study reported increased plasma HexCer levels in patients with critical COVID-19 disease compared with healthy controls [17]. In that study, HexCer levels in asymptomatic COVID-19 patients were comparable to those of healthy controls [17].

Cholesterol, which is transported in circulating low-density lipoprotein (LDL) and high-density lipoprotein (HDL), is frequently reduced in critically ill patients [4,6,18,19]. In human serum, HexCer is predominantly associated with LDL and HDL particles, with comparable distributions of total HexCer and individual HexCer species between these lipoproteins [20]. Collectively, these findings would predict a decline in circulating HexCer levels in patients with severe infectious diseases.

Notably, patients with COVID-19–associated sepsis exhibited higher circulating cholesterol levels than those with sepsis of other etiologies [21]. Similarly, serum cholesterol levels in patients with severe COVID-19 were higher than in patients with cardiogenic shock, suggesting that this alteration is specific to COVID-19–related disease [22].

Underlying liver cirrhosis is an important confounding variable to consider when evaluating lipids. Patients with cirrhosis often exhibit reduced lipoprotein levels and lower serum cholesterol concentrations [23]. Indeed, plasma ceramide species levels, which are primarily associated with LDL [24], were lower in patients with sepsis and liver cirrhosis than in those without this condition [12]. Hexosylceramides are derived from ceramides by glycosylation catalyzed by glucosylceramide synthase [13], suggesting that their levels may also be reduced in cirrhosis. Notably, plasma levels of HexCer d18:1/12:0, d18:1/16:0, and d18:1/22:0 were increased in patients with chronic hepatitis C virus infection who had higher fibrosis scores (>2) relative to those with less advanced fibrosis [24]. Similarly, patients with primary sclerosing cholangitis had elevated serum levels of HexCer18:1;O2/16:0 and HexCer24:1 compared with healthy controls [25].

The present study evaluated plasma levels of five HexCer species in control subjects and in patients with SIRS, sepsis, or septic shock of different etiologies. The primary objective was to compare HexCer profiles between patients with COVID-19-associated sepsis and those with non-COVID-19 sepsis. In addition, we examined HexCer levels in relation to underlying liver cirrhosis and investigated their associations with survival outcomes.

## 2. Materials and Methods

### 2.1. Study Cohort

Ethylenediaminetetraacetic acid plasma samples were obtained from patients treated in the medical intensive care unit (ICU) at the University Hospital Regensburg between August 2018 and January 2024. The ICU specializes in managing patients with liver, gastrointestinal, and infectious diseases. Patients were categorized as having SIRS, sepsis, or septic shock based on the SIRS criteria and the Sepsis-3 definitions [26,27]. Samples from patients with COVID-19 were collected from October 2020 to January 2023.

The median SOFA score was 8 (range, 4–12) in the sepsis group and 18 (range, 13–24) in the septic shock group. The median APACHE II score was 23 (range, 14–28) for patients with sepsis and 58 (range, 44–67) for those with septic shock. At the time of sample collection, patients with septic shock received a median norepinephrine dose of 0.6 µg/kg/min and a median vasopressin dose of 0.05 units/min.

Among invasively ventilated patients, the median Horowitz index (PaO_2_/FiO_2_ ratio) was 138 mmHg (range, 56–234). Patients who required renal replacement therapy were classified as having stage 3 acute kidney injury according to the Kidney Disease: Improving Global Outcomes (KDIGO) Classification System.

The study excluded individuals with multidrug-resistant pathogens, hepatitis virus infections, or HIV infection. Patients who met the predefined eligibility criteria and provided written informed consent, either directly or through an authorized representative, were subsequently included in the retrospective review. Blood samples were collected 12–24 h after ICU admission. Based on the patients’ medical history, symptoms had been present for a median of two days before admission.

Patients were categorized according to ICU outcome, with deaths occurring during the ICU admission defined as non-survivors and patients discharged alive classified as survivors.

Statin use was documented for 47 patients, of whom 4 took statins. In this small subcohort, statins were not associated with changes in HexCer levels (*p* > 0.05).

Clinical laboratory data were retrieved from the Institute of Clinical Chemistry and Laboratory Medicine, while microbiological findings were obtained from the Institute of Clinical Microbiology and Hygiene at our University Hospital.

Twenty-three healthy donors (10 males, 13 females; mean age 42 years, range 25–78), who were employees or students of our hospital and relatives of staff or students, also provided plasma samples. These controls were healthy and of normal body weight; laboratory parameters were not obtained.

Serum from patients with SARS-CoV-2 infection, distinct from the cohort described above, was collected during hospitalization between April 2020 and January 2024. All patients aged ≥18 years who provided informed consent were included in the study. Among these patients, 41 exhibited symptoms indicative of SIRS [26,28] but did not require intensive care and were classified as having moderate COVID-19. Severe COVID-19 was diagnosed in 61 patients who required admission to the ICU [27]. The control group consisted of serum samples from 18 patients hospitalized for various non–COVID-19–related conditions during the same period as the collection of samples from SARS-CoV-2–infected patients.

### 2.2. Quantification of Serum/Plasma HexCer Species

Lipids were obtained from 10 µL of plasma (cohort 1, patients with different disease etiologies) or serum (cohort 2, patients with COVID-19) following the protocol of Bligh and Dyer [29]. Deuterated HexCer 18:1;O2[D5]/18:0 (Avanti Polar Lipids, Alabaster, AL, USA) was added as an internal standard prior to extraction. A fraction of the vacuum-dried chloroform phase was reconstituted in a methanol/chloroform mixture (3:1, *v*/*v*) containing 7.5 mM ammonium acetate (Merck, Darmstadt, Germany; Roth, Karlsruhe, Germany) [30]. HexCer species were analyzed using direct flow injection analysis coupled with tandem mass spectrometry (FIA-MS/MS) on a triple quadrupole mass spectrometer operated in positive ion mode, using a fragment ion of *m*/*z* 264 specific for sphingosine-based lipids. Both precursor ions [M + H]^+^ and the in-source fragment [M + H−H_2_O]^+^ were recorded. The sum of the two transitions was used for quantification, as described for ceramides [30]. Type-II correction was performed, as previously described [31]. A methodological consideration is that the measurements captured hexosylceramide species as a whole and did not differentiate between individual glycosylceramide subclasses.

### 2.3. Statistical Analysis

Hexosylceramide species did not have a normal distribution (Shapiro–Wilk test, *p* < 0.001 for the five HexCer species). Box plots were used to visualize data distribution, including the median, lower and upper quartiles, and extreme values. Individual outliers are indicated by asterisks or circles, and summary statistics (median, minimum, and maximum) are provided separately in tabular format. Statistical analyses were performed using IBM SPSS Statistics 31.0.0.0 (IBM Corp., Armonk, NY, USA; released 2019, updated 2025). The following tests were used to assess relationships between variables:

1. Mann–Whitney U test for comparisons between two groups.
2. Kruskal–Wallis test for comparison of three or more groups.
3. Chi-squared test for categorical variables.
4. Spearman’s correlation for associations between continuous variables.

The data were not corrected for multiple comparisons. This study used lipidomic data in which hundreds of species were measured simultaneously. Data for some of these lipid classes have been published previously [12,32]. Adjusting for all these species and comparisons will prevent the identification of any differences. True effects may fail to reach statistical significance, increasing the Type II error rate, and small but meaningful effects may be missed after correction.

A two-sided *p*-value < 0.05 was considered statistically significant.

## 3. Results

### 3.1. Hexosylceramide Species of Controls and Patients with SIRS, Sepsis, and Septic Shock

This study measured five hexosylceramide species—HexCer 18:1;O2/16:0, 22:0, 23:0, 24:0, and 24:1—in the plasma of 159 patients with SIRS (*n* = 39), sepsis (*n* = 41), or septic shock (*n* = 79), as well as in 23 healthy controls. It has to be stated that hexosylceramide species, rather than specific glycosylceramide subclasses, have been measured. The patient cohort included 48 females and 111 males, whereas the control group included 13 females and 10 males (*p* = 0.013).

Despite similar concentrations of procalcitonin, C-reactive protein, and white blood cells across disease categories, immature granulocytes were selectively elevated in patients with sepsis and septic shock relative to those presenting with SIRS (Table 1). Bilirubin, albumin, aminotransferase, and gamma-glutamyltransferase levels were similar across groups (Table 1). Body mass index was higher in patients with septic shock than in those with SIRS (Table 1). Need for vasopressor therapy, dialysis and ventilation increased with disease severity (Table 1).

Male and female patients had comparable plasma levels of all HexCer species (*p* > 0.05 for all species) and total HexCer levels (*p* = 0.589). In the control cohort, females exhibited higher HexCer 18:1;O2/16:0 levels than males (*p* = 0.042), while the remaining HexCer species (*p* > 0.05 for all) and total HexCer levels (*p* = 0.208) did not differ between sexes.

**Figure 1:**
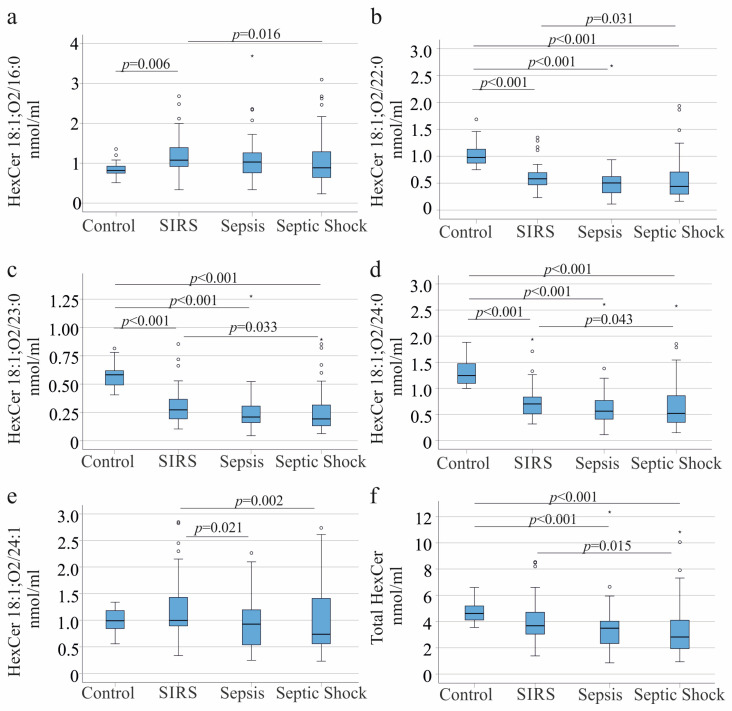
Hexosylceramide (HexCer) species in plasma of controls, patients with systemic inflammatory response syndrome (SIRS), sepsis, or septic shock. (**a**) HexCer18:1;O2/16:0; (**b**) HexCer18:1;O2/22:0; (**c**) HexCer18:1;O2/23:0; (**d**) HexCer18:1;O2/24:0; (**e**) HexCer18:1;O2/24:1; and (**f**) total HexCer levels in plasma of controls, patients with SIRS, sepsis, or septic shock. Outliers are represented by circles and small asterisks.

HexCer 18:1;O2/16:0 levels were elevated in patients with SIRS compared with controls, but did not differ among sepsis, septic shock, and controls. Levels were lower in septic shock compared to SIRS (Figure 1a). HexCer 18:1;O2/22:0, 18:1;O2/23:0, and 18:1;O2/24:0 were reduced in all patient groups compared with controls and were also lower in septic shock compared to SIRS (Figure 1b–d). HexCer 18:1;O2/24:1 was higher in SIRS than in sepsis and septic shock (Figure 1e). Total HexCer levels were lower in sepsis and septic shock than in controls, who had levels similar to those of patients with SIRS. Levels were lower in septic shock than in SIRS (Figure 1f).

Because HexCer species levels showed sex-specific differences and the sexes differed between patients and controls, a sex-specific analysis was also conducted. In females, HexCer 18:1;O2/16:0 and 24:1 did not differ between controls and patients, regardless of disease severity (Figure S1a,e). HexCer 18:1;O2/22:0, 18:1;O2/23:0, and 18:1;O2/24:0 were reduced in all patient groups compared with controls (Figure S1b–d). Total HexCer levels were lower in patients with sepsis and septic shock than in controls, who had levels similar to those in patients with SIRS (Figure S1f).

In males, HexCer 18:1;O2/16:0 levels were higher in SIRS, sepsis, and septic shock compared to the controls (Figure S2a). HexCer 18:1;O2/22:0, 18:1;O2/23:0, and 18:1;O2/24:0 were reduced in all patient groups compared with controls (Figure S2b–d). HexCer 18:1;O2/22:0, 24:0, 24:1, and total HexCer levels were lower in septic shock than in SIRS (Figure S2b,d–f). Total HexCer levels were lower in patients with septic shock than in controls (Figure S2f).

This analysis showed that the association of HexCer species levels is not greatly different between the sexes, and here the comparatively low number of females may have prevented the identification of significant changes observed in males.

### 3.2. Hexosylceramide Species and Liver Cirrhosis

**Figure 2:**
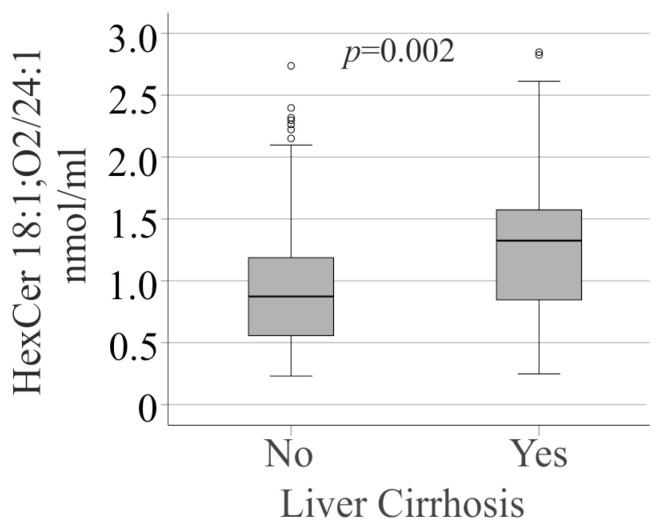
HexCer18:1;O2/24:1 of patients with and without liver cirrhosis. Outliers are represented by circles.

Plasma HexCer 18:1;O2/24:1 was different between patients with and without liver cirrhosis, with lower levels observed in the non-cirrhotic cohort (Figure 2).

After excluding the 31 patients with liver cirrhosis, HexCer 18:1;O2/24:1 remained lower in patients with septic shock compared with those with SIRS (*p* = 0.045) and was comparable to levels in controls and sepsis patients.

Several ceramide species are reduced in patients with cirrhosis and SIRS/sepsis, as recently analyzed in this cohort [12]. As a consequence, the HexCer/ceramide ratios for species 16:0 (1.1 (0.3–2.5) and 1.9 (0.9–5.7), *p* < 0.001), 22:0 (0.3 (0.1–1.6) and 1.0 (0.2–3.4), *p* < 0.001), 23:0 (0.2 (0.1–1.1) and 0.8 (0.1–2.4), *p* < 0.001), 24:0 (0.2 (0–0.8) and 0.5 (0.1–2.6), *p* < 0.001), and 24:1 (0.2 (0.1–0.9) and 0.8 (0.1–2.1), *p* < 0.001) were markedly increased in cirrhosis.

The Hex/Cer ratio of patients without cirrhosis was 0.3 (0.1–1.0) and was 0.8 (0.2–2.3) in cirrhosis (*p* < 0.001).

### 3.3. Hexosylceramide Species and SARS-CoV-2 Infection

The cohort included 24 patients with confirmed cases of SARS-CoV-2 infection. These patients had lower levels of procalcitonin, eosinophils, and bilirubin, and higher levels of albumin and cholesterol than patients with sepsis caused by other pathogens (Table S1). The COVID-19 patients had more severe illness and a higher need for vasopressor therapy and ventilation (Table S1).

**Figure 3:**
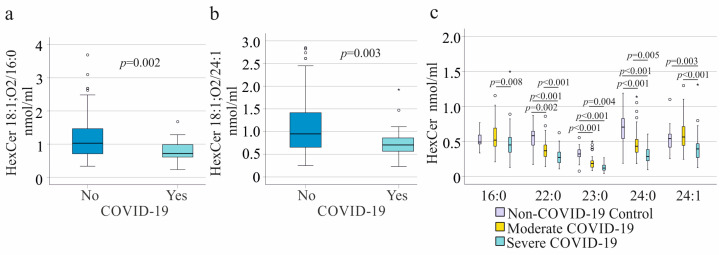
Hexosylceramide (HexCer) species in plasma/serum of patients with SARS-CoV-2 infection. (**a**) Plasma HexCer 18:1;O2/16:0 levels in patients with sepsis caused by SARS-CoV-2 (Yes) or with a different disease etiology (No); (**b**) Plasma HexCer 18:1;O2/24:1 of patients with sepsis caused by SARS-CoV-2 or with a different disease etiology; (**c**) HexCer species in the serum of non-COVID-19 patients (purple boxes), COVID-19 patients with moderate disease (yellow boxes) and severe COVID-19 cases (green boxes). Outliers are represented by small circles and asterisks.

The 24 patients with SARS-CoV-2 infection had lower levels of HexCer 18:1;O2/16:0 and 18:1;O2/24:1, as well as lower total serum HexCer levels (*p* = 0.039), compared with patients with non–SARS-CoV-2 infections (Figure 3a,b).

Serum HexCer levels were also analyzed across 18 patients with non–COVID-19 diseases, 41 with moderate COVID-19, and 61 with severe COVID-19 (Table 2). The non-COVID-19 patients were younger, had lower serum C-reactive protein levels, and had higher cholesterol levels. Patients with severe COVID-19 had the highest body mass index, C-reactive protein, and procalcitonin levels, and the lowest albumin levels (Table 2). Interleukin-6 levels did not differ between moderate and severe cases (Table 2).

All HexCer species were reduced in severe compared to moderate COVID-19 (Figure 3c). Notably, HexCer 18:1;O2/22:0, 23:0, and 24:0 were already low in patients with moderate COVID-19 compared with non–COVID-19 patients (Figure 3c). Whereas HexCer 18:1;O2/24:1 was lower in patients with severe COVID-19 compared to controls, this difference was not significant for HexCer 18:1;O2/16:0 (Figure 3c).

### 3.4. Hexosylceramide Species in the Subgroups of Patients Requiring Ventilation or Septic Shock

Because all patients with SARS-CoV-2 were ventilated (Table S1), plasma HexCer levels were also compared with those of sepsis patients with other disease etiologies who also required ventilation (73 patients). COVID-19 patients had lower procalcitonin, bilirubin, and eosinophil counts, and higher albumin levels and total cholesterol levels (Table S2). HexCer 18:1;O2/16:0 (*p* = 0.027) was lower in COVID-19, while other species and total HexCer levels did not differ between the cohorts (*p* > 0.05). Mortality of the two cohorts was similar (*p* = 0.526).

When patients with septic shock (58 non-COVID-19 and 21 COVID-19 patients) were compared, HexCer species levels did not differ between the cohorts, while procalcitonin (*p* = 0.012), eosinophil number (*p* = 0.001), and bilirubin (*p* = 0.029) were lower in COVID-19 patients, and total cholesterol (*p* = 0.017) and albumin (*p* = 0.002) were higher. Mortality of the two cohorts was similar (*p* = 0.380).

When only female patients with septic shock (13 non-COVID-19 and 5 COVID-19) were compared, HexCer species levels did not differ between cohorts, whereas procalcitonin was lower in COVID-19 (*p* = 0.035). When only male patients with septic shock (45 non-COVID-19 and 16 COVID-19 patients) were included, HexCer species levels did not differ between the cohorts, while eosinophil count (*p* = 0.005) and bilirubin (*p* = 0.019) were lower, and cholesterol (*p* = 0.008) and albumin (*p* = 0.005) were higher in COVID-19.

### 3.5. Correlations of Hexosylceramide Species with Inflammation and Markers of Liver Disease

Correlation analyses were performed after excluding patients with liver cirrhosis. In the remaining 128 patients, neither individual HexCer species nor total HexCer levels correlated with age or BMI. No significant correlations were observed with C-reactive protein or procalcitonin. Leukocyte count positively correlated with HexCer 18:1;O2/24:1 (r = 0.216, *p* = 0.015) and total HexCer levels (r = 0.178, *p* = 0.046). Bilirubin and aminotransferase levels were not associated with HexCer concentrations. However, total HexCer levels (r = 0.307, *p* = 0.002) and individual species—HexCer 18:1;O2/16:0 (r = 0.268, *p* = 0.007), 18:1;O2/22:0 (r = 0.336, *p* = 0.001), 18:1;O2/23:0 (r = 0.372, *p* < 0.001), 18:1;O2/24:0 (r = 0.310, *p* = 0.002), and 18:1;O2/24:1 (r = 0.272, *p* = 0.006)—positively correlated with gamma-glutamyltransferase. HexCer 18:1;O2/16:0 (r = −0.224, *p* = 0.016) negatively correlated with albumin. Total HexCer levels (r = 0.590, *p* < 0.001) and individual species—HexCer 18:1;O2/16:0 (r = 0.462, *p* < 0.001), 18:1;O2/22:0 (r = 0.657, *p* < 0.001), 18:1;O2/23:0 (r = 0.644, *p* < 0.001), 18:1;O2/24:0 (r = 0.667, *p* < 0.001), and 18:1;O2/24:1 (r = 0.499, *p* < 0.001)—positively correlated with serum cholesterol levels. Gamma-glutamyltransferase (r = 0.368, *p* < 0.001), but not bilirubin, positively correlated with total cholesterol levels.

### 3.6. Hexosylceramide Species and Survival

**Figure 4:**
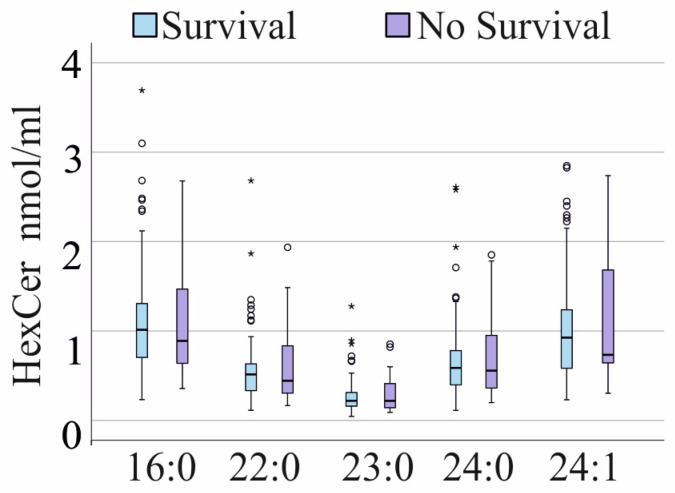
Hexosylceramide (HexCer) species of patients who survived and those who died. HexCer species in the plasma of the patients who survived and those who did not survive were similar. Outliers are represented by small circles and asterisks.

The 38 non-survivors exhibited plasma levels of all HexCer species comparable to those of survivors (*p* > 0.05 for all comparisons; Figure 4). These findings remained unchanged after excluding patients with COVID-19 or liver cirrhosis (*p* > 0.05 for all comparisons).

## 4. Discussion

Here we present the concept that critical illness is associated with distinct alterations in HexCer levels that are similar in COVID-19-associated sepsis and non-COVID-19 sepsis and do not differ greatly between sexes.

Plasma HexCer 18:1;O2/22:0, 23:0, and 24:0 are similarly reduced in patients with SIRS, sepsis, or septic shock compared with healthy controls. These species are also lower in septic shock compared to SIRS. HexCer 18:1;O2/16:0 and 18:1;O2/24:1 differ between patients with SIRS and septic shock, and the latter species is also lower in sepsis than in SIRS. However, these differences between patients with SIRS and septic shock were too small for diagnostic purposes. HexCer 18:1;O2/16:0 is higher in SIRS than in controls, and this was also significant in males, though a similar trend was noticed in females, suggesting that it is upregulated in less severe disease. Again, this difference was small and is not of diagnostic value.

In our study cohort, CRP, procalcitonin, and white blood cell count did not differ among patients with SIRS, sepsis, and septic shock. A previous study also showed that CRP and procalcitonin were similar between patients with sepsis and those with septic shock on day 1 of hospital admission [33]. Another study also reported on similar levels in patients with sepsis or septic shock [34]. Baseline CRP levels may even be in the normal range in patients with sepsis [35]. There was no significant association between procalcitonin levels and disease severity assessed by the APACHE II and SOFA scores [36]. Moreover, leukocyte counts are not specific for sepsis severity [37]. These studies and our analysis show that the common clinical markers of inflammation cannot be used to assess disease severity in patients with SIRS, sepsis, or septic shock. HexCer species and total HexCer levels were consistently reduced in patients with septic shock compared with those with SIRS, indicating that their serum concentrations are more closely associated with disease severity than CRP or procalcitonin. However, because the observed differences were small, HexCer levels are unlikely to be useful as a prognostic biomarker.

HexCer 18:1;O2/22:0, 23:0, and 24:0 are consistently and strongly decreased in plasma from patients with SIRS, sepsis, or septic shock. These species are already reduced in patients with moderate COVID-19 and change only weakly in severe disease. This pattern suggests that the decline in these HexCer species occurs early in the disease process. Whether these changes are solely a consequence of systemic inflammation or also contribute to disease pathogenesis remains to be investigated.

HexCers are a comparatively understudied class of lipids derived from ceramides via glycosylation [13]. Notably, HexCer levels do not simply mirror changes in their corresponding ceramide precursors. While plasma ceramide 18:1;O2/22:0 levels are increased, ceramide 18:1;O2/23:0 and 18:1;O2/24:0 are decreased in sepsis compared to the controls [12]. Furthermore, ceramide 18:1;O2/16:0 and 18:1;O2/24:1 are elevated in sepsis, whereas the corresponding HexCer species remain unchanged. These findings indicate that HexCer metabolism is regulated independently of ceramide abundance, rather than passively reflecting ceramide levels.

Patients with severe SARS-CoV-2 infection had lower levels of procalcitonin, eosinophils, and bilirubin, and higher levels of cholesterol and albumin than patients with septic shock caused by other pathogens. These differences persisted when ventilated patients with and without SARS-CoV-2 infection were compared.

Procalcitonin was found to be reduced in viral sepsis compared with bacterial sepsis, suggesting that the lower levels observed in COVID-19 may be explained by this [38]. A comparison of severe COVID-19 and non-COVID-19 patients on day 1 revealed similar eosinophil counts but higher platelet and CRP levels in COVID-19 patients [39]. Another study compared bacterial and SARS-CoV-2 infections and found lower levels of IL-6, procalcitonin, and CRP in the latter [40]. At the time of ICU admission, patients who died from COVID-19, when compared with those who died with sepsis from other causes, exhibited lower eosinophil counts and reduced levels of procalcitonin, IL-6, total and direct bilirubin, but similar CRP and albumin levels [41]. Currently, procalcitonin is often assumed to be lower in COVID-19 than in non-COVID-19 sepsis, whereas the further differences described above are not consistent across studies. Evaluation of COVID-19-specific biomarkers requires comparisons with non-COVID-19 patients of comparable disease severity and validation in large cohorts.

HexCer 18:1;O2/16:0 species levels of patients with SARS-CoV-2 infection requiring ventilation were lower compared to ventilated non-COVID-19 patients. Among patients with septic shock, no significant differences in HexCer species concentrations were observed between the two groups. However, compared with the non-COVID-19 cohort, patients with COVID-19 had significantly lower procalcitonin, eosinophil counts, and bilirubin levels, whereas total cholesterol and albumin concentrations were significantly higher. This indicates that none of these measures are related to disease severity, as assessed by clinic scores, since all patients had septic shock.

Glycosphingolipids play a critical role in viral infection and propagation. Blocking glucosylceramide synthase activity impairs SARS-CoV-2 propagation in Vero E6 cells, which originate from African green monkey kidney cells [42]. SARS-CoV-2 infection of these cells results in increased intracellular levels of multiple HexCer species, including HexCer 16:0, 20:0, 23:0, 24:0, and 24:1 [14]. HexCer 16:0 and 22:0 in mouse serum were induced at day 5 postinfection [14]. These experimental findings contrast with observations in COVID-19 patients, in whom serum HexCer levels are reduced relative to healthy controls [15]. Consistent with this previous report, four out of five HexCer species analyzed in the present cohort were lower in patients with COVID-19 sepsis than in healthy controls. This discrepancy between experimental infection models and clinical disease suggests fundamental differences in HexCer regulation between systemic inflammatory responses in rodents and humans.

Uranbileg et al. directly compared non–COVID-19 and COVID-19 patients and reported lower serum HexCer levels in the former group [15]. Across these comparative analyses, patients with non-COVID infectious diseases exhibited greater disease severity than those with COVID-19 [15]. Our analysis shows comparable plasma HexCer levels in patients with septic shock of COVID-19 and non–COVID-19 etiology. The greater disease severity observed in the non-COVID-19 cohort in the study by Uranbileg [15] may have contributed to the differences in HexCer levels between patients with non-COVID-19 infections and those with COVID-19. However, direct comparison between these studies is limited by analytical differences. Uranbileg et al. quantified seven HexCer species [15], of which only HexCer 16:0 and 22:0 overlap with those measured in the present study. In addition to HexCer 22:0, HexCer 24:0 and 24:1 are among the most abundant HexCer species in human plasma [20] and were included in our analysis but not assessed by Uranbileg et al. [15]. Given these differences in lipid coverage, direct comparison of absolute HexCer levels between the two studies is not recommended. In our cohort, HexCer levels did not differ between COVID-19 and non-COVID-19 patients, all of whom had septic shock. Whether differences in disease severity between the cohorts exist that are not adequately captured by current severity stratification scores remains to be determined.

Plasma cholesterol levels were higher in septic patients with concomitant SARS-CoV-2 infection than in those with sepsis from non-COVID-19 causes [21]. In addition, several lysophosphatidylcholine species are increased in COVID-19 compared with non-COVID-19 sepsis [32,43]. This supports the concept that critical illness induces distinct, etiology-specific alterations in lipid metabolism between COVID-19 and non-COVID-19 sepsis, which do not translate to all lipid classes, as HexCer and ceramide levels [12] are similar between these patients.

Plasma HexCer levels did not correlate with markers of inflammation, immune cell populations, or measures of liver disease. There was a positive correlation of all HexCer species with GGT. Median GGT levels exceeded the normal cutoff, but they did not differ among patients with SIRS, sepsis, or septic shock. HexCer species did not correlate with bilirubin, a further marker of cholangitis [44], excluding a close association of HexCer levels in blood with biliary disease. GGT was positively correlated with cholesterol, and all HexCer species were also positively associated with cholesterol, suggesting that the association between HexCer species and GGT may, at least in part, be explained by their shared relationship with cholesterol.

Elevated HexCer levels are described in patients with advanced liver disease. Specifically, increased plasma concentrations of HexCer d18:1/12:0, 16:0, and 22:0, together with unchanged levels of HexCer 24:0 and 24:1, have been described [24]. In our cohort of sepsis patients, HexCer 18:1;O2/24:1 was significantly increased in individuals with liver cirrhosis. Whether concomitant sepsis modifies the HexCer pattern typically associated with cirrhosis requires further investigation.

Notably, several ceramide species are reduced in liver cirrhosis [45] and were likewise decreased in patients with cirrhosis and sepsis in our cohort [12]. As a consequence, the HexCer/ceramide ratios for species 16:0, 22:0, 23:0, 24:0, and 24:1 were markedly increased. A reduced HexCer/ceramide ratio has been associated with lower hepatic triglyceride accumulation [46], whereas an elevated ratio has been linked to increased cardiovascular risk [47]. Whether this pronounced imbalance in HexCer/ceramide ratios has pathological relevance in sepsis patients with liver cirrhosis remains to be determined.

Several limitations should be acknowledged. Laboratory measurements were not available for the control group, limiting comparisons between patients and controls. Furthermore, the controls were younger than the patients, which may have confounded our findings. However, the concentrations of HexCer species did not correlate with age, suggesting that age differences are unlikely to account for the observed results. Since sampling was restricted to the time of hospital admission, the dynamics of HexCer alterations over the progression of illness could not be investigated. In addition, the duration and trajectory of illness prior to ICU admission were not documented, precluding analysis of pre-ICU disease dynamics. As an observational study, this work cannot determine when systemic HexCer levels decline during disease progression. Such reductions may occur early in the disease course, during acute illness, or as a consequence of therapeutic interventions and/or medication use. Plasma and serum samples were obtained not concurrently in the fasted state, and HexCer 18:1, O2/14:0, and 16:0 levels are lower in fasted compared to non-fasted serum and EDTA plasma [48]. Hexosylceramide species, rather than specific glycosylceramide subclasses, have been measured, and analysis of these subclasses may provide further insight into the complex association of lipid species with sepsis.

## 5. Conclusions

Together, these findings indicate that critical illness is associated with distinct alterations in HexCer species levels. Specifically, circulating levels of HexCer 18:1;O2/22:0, 23:0, and 24:0 were strongly reduced in patients with SIRS, with a modest further decline observed in patients with more severe disease. HexCer 18:1, O2/16:0, and 24:1 are decreased in septic shock, but this effect is too small for diagnostic purposes.

## Figures and Tables

**Table 1:** Clinical details of patients with systemic inflammatory response syndrome (SIRS), sepsis, and septic shock. Superscript numbers indicate the number of patients included in the analysis when specific variables were available only for a subset of the cohort. Data are presented as median (minimum–maximum). Statistical tests used: Kruskal–Wallis test and Chi-squared test. The identical *p*-values in the respective columns indicate significant differences.

Parameters	SIRS	Sepsis	Septic Shock
Males/Females	27/12 (*n* = 39)	23/18 (*n* = 41)	61/18 (*n* = 79)
Age, years	59 (29–88)	58 (28–81)	61 (21–93)
Body mass index, kg/m^2^	24.4 (18.3–51.4) ^37,^ *^p^* ^= 0.031^	26.4 (18.4–54.5) ^40^	28.7 (15.4–55.6) *^p^* ^= 0.031^
C-reactive protein, mg/L	145 (12–402)	130 (28–503)	164 (18–697)
Procalcitonin, ng/mL	1.04 (0.05–270.00) ^78^	0.6 (0.06–112.27) ^39^	1.84 (0.08–114.40)
Leukocytes, *n*/nL	10.30 (0.06–37.38)	10.98 (0.28–34.17)	10.20 (0.32–1586.00)
Neutrophils, *n*/nL	6.33 (1.46–29.73) ^36^	7.42 (0–70.20) ^39^	8.50 (0–48.40) ^78^
Basophils, *n*/nL	0.04 (0–0.38) ^36^	0.04 (0–0.90) ^39^	0.04 (0–0.60)
Eosinophils, *n*/nL	0.16 (0–2.89) ^36^	0.06 (0–1.75) ^39^	0.12 (0–8.80)
Monocytes, *n*/nL	0.66 (0.02–3.59) ^36^	0.95 (0.08–45.00) ^39^	0.71 (0–10.90)
Lymphocytes, *n*/nL	0.87 (0.10–2.79) ^36^	1.04 (0.29–16.80) ^39^	0.94 (0.08–28.60)
Immature granulocytes, *n*/nL	0.04 (0.01–0.44) ^35, *p* < 0.001, *p* = 0.029^	0.12 (0–6.19) ^39, *p* = 0.029^	0.22 (0–6.19) *^p^*^ < 0.001^
Total bilirubin, mg/dL	0.85 (0.10–30.50) ^36^	0.60 (0.10–18.10) ^39^	0.80 (0.10–20.10) ^73^
Albumin, g/L	20.8 (13.0–32.8) ^34^	23.5 (15.5–41.9) ^39^	23.2 (6.3–42.0) ^74^
Aspartate aminotransferase, U/L	46 (6–1562) ^36^	41 (8–603) ^37^	48 (8–1597) ^71^
Alanine aminotransferase, U/L	34 (8–288) ^36^	30 (7–559) ^35^	32 (6–770) ^71^
Gamma-glutamyl transferase, U/L	180 (23–1093) ^33^	142 (25–467) ^35^	95 (11–1266) ^59^
Cholesterol nmol/mL	2384 (906–6223)	2367 (712–8529)	2207 (904–6840)
Vasopressor therapy	3 *^p^* ^< 0.001^	18 *^p^* ^< 0.001^	75 *^p^* ^< 0.001^
Dialysis	1 *^p^* ^< 0.001^	5 *^p^* ^< 0.001^	48 *^p^* ^< 0.001^
Ventilation	4 *^p^* ^< 0.001^	18 *^p^* ^< 0.001^	75 *^p^* ^< 0.001^

**Table 2:** Clinical details of patients with and without SARS-CoV-2. Data are given as median (minimum–maximum). Superscript numbers refer to the number of patients for whom this data was documented, as not all patients were included in the data collection. Statistical tests used: Kruskal–Wallis test and Chi-squared test. The identical *p*-values in the respective columns indicate significant differences.

Parameters	Non-COVID-19 Patients	Moderate COVID-19	Severe COVID-19
Males/Females	8/10	23/18	43/18
Age, years	48 (27–70) ^*p* = 0.009, *p* = 0.003^	60 (22–83) ^*p* = 0.003^	57 (31–83) ^*p* = 0.009^
Body mass index, kg/m^2^	Not defined	26.2 (18.4–44.6) ^23, *p* = 0.006^	29.4 (19.2–66.7) ^57, *p* = 0.006^
C-reactive protein, mg/L	3 (0–40) ^13, *p* < 0.001^	25 (0–218) *^p^* ^< 0.001, *p* = 0.016^	73 (1–367) ^*p* < 0.001, *p* = 0.016^
Procalcitonin, ng/mL	Not defined	0.09 (0.00–25.00) ^31, *p* < 0.001^	0.24 (0.06–367.00) *^p^* ^< 0.001^
Interleukin-6, pg/mL	Not defined	30 (4–265) ^22^	35 (3–1175)
Albumin, g/L	Not defined	33.5 (18.9–41.0) ^25, *p* < 0.001^	27.2 (19.3–30.0) ^*p* < 0.001^
Cholesterol nmol/mL	5051 (1963–8674) *^p^* ^< 0.001, *p* = 0.001^	3132 (1936–7802) *^p^* ^= 0.001^	3178 (1611–5590) *^p^* ^< 0.001^

## Data Availability

Data are shown in the manuscript. Original data can be obtained from the corresponding author.

## Supplementary Materials

The following supporting information can be downloaded at: https://www.mdpi.com/article/10.3390/biomedicines14071635/s1, Figure S1. Hexosylceramide (HexCer) species in plasma of female controls, patients with systemic inflammatory response syndrome (SIRS), sepsis, or septic shock. (**a**) HexCer18:1;O2/16:0; (**b**) HexCer18:1;O2/22:0; (**c**) HexCer18:1;O2/23:0; (**d**) HexCer18:1;O2/24:0; (**e**) HexCer18:1;O2/24:1; (**f**) total HexCer levels in plasma of controls, patients with SIRS, sepsis, or septic shock. Outliers are represented by circles and small asterisks; Figure S2. Hexosylceramide (HexCer) species in plasma of male controls, patients with systemic inflammatory response syndrome (SIRS), sepsis, or septic shock. (**a**) HexCer18:1;O2/16:0; (**b**) HexCer18:1;O2/22:0; (**c**) HexCer18:1;O2/23:0; (**d**) HexCer18:1;O2/24:0; (**e**) HexCer18:1;O2/24:1; (**f**) total HexCer levels in plasma of controls, patients with SIRS, sepsis, or septic shock. Outliers are represented by circles and small asterisks; Table S1. Clinical details of patients with systemic inflammatory response syndrome (SIRS), sepsis, and septic shock. Numbers in superscript refer to patients for whom these data were available when data were not collected from the entire cohort. Data are presented as median (minimum–maximum). Statistical tests used: Kruskal-Wallis test and Chi-squared test; Table S2. Characteristics of patients with non-COVID-19 and COVID-19 sepsis requiring ventilation. Numbers in superscript refer to patients for whom these data were available when data were not collected from the entire cohort. Data are presented as median (minimum–maximum). Statistical tests used: Kruskal-Wallis test and Chi-squared test.

## Abbreviations

The following abbreviations are used in this manuscript:

ALT	Alanine aminotransferase
AST	Aspartate aminotransferase
GGT	Gamma-glutamyl transferase
HexCer	Hexosylceramide
Interleukin	IL
SARS-CoV-2	Severe acute respiratory syndrome coronavirus 2
SIRS	Systemic inflammatory response syndrome

## References

[1] Barichello T., Generoso J.S., Singer M., Dal-Pizzol F. (2022). Biomarkers for sepsis: More than just fever and leukocytosis-a narrative review. Crit. Care.

[2] He R.R., Yue G.L., Dong M.L., Wang J.Q., Cheng C. (2024). Sepsis Biomarkers: Advancements and Clinical Applications-A Narrative Review. Int. J. Mol. Sci..

[3] Konjety P., Chakole V.G. (2024). Beyond the Horizon: A Comprehensive Review of Contemporary Strategies in Sepsis Management Encompassing Predictors, Diagnostic Tools, and Therapeutic Advances. Cureus.

[4] Barker G., Leeuwenburgh C., Brusko T., Moldawer L., Reddy S.T., Guirgis F.W. (2021). Lipid and Lipoprotein Dysregulation in Sepsis: Clinical and Mechanistic Insights into Chronic Critical Illness. J. Clin. Med..

[5] Harris H.W., Gosnell J.E., Kumwenda Z.L. (2000). The lipemia of sepsis: Triglyceride-rich lipoproteins as agents of innate immunity. J. Endotoxin Res..

[6] Hofmaenner D.A., Arina P., Kleyman A., Page Black L., Salomao R., Tanaka S., Guirgis F.W., Arulkumaran N., Singer M. (2023). Association Between Hypocholesterolemia and Mortality in Critically Ill Patients with Sepsis: A Systematic Review and Meta-Analysis. Crit. Care Explor..

[7] Gomez-Munoz A., Presa N., Gomez-Larrauri A., Rivera I.G., Trueba M., Ordonez M. (2016). Control of inflammatory responses by ceramide, sphingosine 1-phosphate and ceramide 1-phosphate. Prog. Lipid Res..

[8] Gaggini M., Ndreu R., Michelucci E., Rocchiccioli S., Vassalle C. (2022). Ceramides as Mediators of Oxidative Stress and Inflammation in Cardiometabolic Disease. Int. J. Mol. Sci..

[9] Chung H.Y., Claus R.A. (2020). Keep Your Friends Close, but Your Enemies Closer: Role of Acid Sphingomyelinase During Infection and Host Response. Front. Med..

[10] Amalia L., Tsai S.L. (2023). Ceramide’s Role and Biosynthesis: A Brief Review. Biotechnol. Bioprocess Eng..

[11] Drobnik W., Liebisch G., Audebert F.X., Frohlich D., Gluck T., Vogel P., Rothe G., Schmitz G. (2003). Plasma ceramide and lysophosphatidylcholine inversely correlate with mortality in sepsis patients. J. Lipid Res..

[12] Pavel V., Mester P., Höring M., Krautbauer S., Liebisch G., Schmid S., Müller M., Buechler C. (2026). A Reproducible Ceramide Phenotype of Sepsis Across Aetiologies—A Monocenter Cohort Study. J. Inflamm. Res..

[13] Zhao X., Pandey M.K. (2025). Central Roles of Glucosylceramide in Driving Cancer Pathogenesis. Int. J. Mol. Sci..

[14] Vitner E.B., Avraham R., Politi B., Melamed S., Israely T. (2021). Elevation in sphingolipid upon SARS-CoV-2 infection: Possible implications for COVID-19 pathology. Life Sci. Alliance.

[15] Uranbileg B., Isago H., Nakayama H., Jubishi D., Okamoto K., Sakai E., Kubota M., Tsutsumi T., Moriya K., Kurano M. (2024). Comprehensive metabolic modulations of sphingolipids are promising severity indicators in COVID-19. FASEB J..

[16] Torretta E., Garziano M., Poliseno M., Capitanio D., Biasin M., Santantonio T.A., Clerici M., Lo Caputo S., Trabattoni D., Gelfi C. (2021). Severity of COVID-19 Patients Predicted by Serum Sphingolipids Signature. Int. J. Mol. Sci..

[17] Toro D.M., da Silva-Neto P.V., de Carvalho J.C.S., Fuzo C.A., Perez M.M., Pimentel V.E., Fraga-Silva T.F.C., Oliveira C.N.S., Caruso G.R., Vilela A.F.L. (2023). Plasma Sphingomyelin Disturbances: Unveiling Its Dual Role as a Crucial Immunopathological Factor and a Severity Prognostic Biomarker in COVID-19. Cells.

[18] Pirillo A., Catapano A.L., Norata G.D. (2015). HDL in infectious diseases and sepsis. High Density Lipoproteins.

[19] Taylor R., Zhang C., George D., Kotecha S., Abdelghaffar M., Forster T., Santos Rodrigues P.D., Reisinger A.C., White D., Hamilton F. (2024). Low circulatory levels of total cholesterol, HDL-C and LDL-C are associated with death of patients with sepsis and critical illness: Systematic review, meta-analysis, and perspective of observational studies. eBioMedicine.

[20] Scherer M., Bottcher A., Schmitz G., Liebisch G. (2011). Sphingolipid profiling of human plasma and FPLC-separated lipoprotein fractions by hydrophilic interaction chromatography tandem mass spectrometry. Biochim. Biophys. Acta.

[21] Birner C., Mester P., Liebisch G., Horing M., Schmid S., Muller M., Pavel V., Buechler C. (2024). Lipid Metabolism Disorders as Diagnostic Biosignatures in Sepsis. Infect. Dis. Rep..

[22] Schmelter F., Foh B., Mallagaray A., Rahmoller J., Ehlers M., Lehrian S., von Kopylow V., Kunsting I., Lixenfeld A.S., Martin E. (2021). Metabolic and Lipidomic Markers Differentiate COVID-19 From Non-Hospitalized and Other Intensive Care Patients. Front. Mol. Biosci..

[23] van den Berg E.H., Flores-Guerrero J.L., Gruppen E.G., Garcia E., Connelly M.A., de Meijer V.E., Bakker S.J.L., Blokzijl H., Dullaart R.P.F. (2022). Profoundly Disturbed Lipoproteins in Cirrhotic Patients: Role of Lipoprotein-Z, a Hepatotoxic LDL-like Lipoprotein. J. Clin. Med..

[24] Li J.F., Qu F., Zheng S.J., Ren F., Wu H.L., Liu M., Ren J.Y., Chen Y., Duan Z.P., Zhang J.L. (2015). Plasma sphingolipids: Potential biomarkers for severe hepatic fibrosis in chronic hepatitis C. Mol. Med. Rep..

[25] Elger T., Huss M., Liebisch G., Horing M., Loibl J., Kandulski A., Muller M., Tews H.C., Buechler C. (2025). Elevated long-to-very-long-chain ceramide ratio correlates with disease severity in inflammatory bowel disease and primary sclerosing cholangitis. Sci. Rep..

[26] Bone R.C. (1995). Sepsis, sepsis syndrome, and the systemic inflammatory response syndrome (SIRS). Gulliver in Laputa. JAMA.

[27] Singer M., Deutschman C.S., Seymour C.W., Shankar-Hari M., Annane D., Bauer M., Bellomo R., Bernard G.R., Chiche J.D., Coopersmith C.M. (2016). The Third International Consensus Definitions for Sepsis and Septic Shock (Sepsis-3). JAMA.

[28] Bone R.C., Balk R.A., Cerra F.B., Dellinger R.P., Fein A.M., Knaus W.A., Schein R.M., Sibbald W.J. (1992). Definitions for sepsis and organ failure and guidelines for the use of innovative therapies in sepsis. The ACCP/SCCM Consensus Conference Committee. American College of Chest Physicians/Society of Critical Care Medicine. Chest.

[29] Bligh E.G., Dyer W.J. (1959). A rapid method of total lipid extraction and purification. Can. J. Biochem. Physiol..

[30] Liebisch G., Drobnik W., Reil M., Trumbach B., Arnecke R., Olgemoller B., Roscher A., Schmitz G. (1999). Quantitative measurement of different ceramide species from crude cellular extracts by electrospray ionization tandem mass spectrometry (ESI-MS/MS). J. Lipid Res..

[31] Liebisch G., Lieser B., Rathenberg J., Drobnik W., Schmitz G. (2004). High-throughput quantification of phosphatidylcholine and sphingomyelin by electrospray ionization tandem mass spectrometry coupled with isotope correction algorithm. Biochim. Biophys. Acta.

[32] Pavel V., Mester P., Horing M., Liebisch G., Schmid S., Muller M., Buechler C. (2025). Distinct Plasma LPC Signatures Differentiate COVID-19 Sepsis from Other Sepsis Aetiologies. Biomedicines.

[33] Schupp T., Weidner K., Rusnak J., Jawhar S., Forner J., Dulatahu F., Dudda J., Bruck L.M., Hoffmann U., Bertsch T. (2024). C-reactive protein and procalcitonin during course of sepsis and septic shock. Ir. J. Med. Sci..

[34] Aliu-Bejta A., Atelj A., Kurshumliu M., Dreshaj S., Barsic B. (2020). Presepsin values as markers of severity of sepsis. Int. J. Infect. Dis..

[35] Wasserman A., Karov R., Shenhar-Tsarfaty S., Paran Y., Zeltzer D., Shapira I., Trotzky D., Halpern P., Meilik A., Raykhshtat E. (2019). Septic patients presenting with apparently normal C-reactive protein: A point of caution for the ER physician. Medicine.

[36] Durrance R.J., Ullah T., Patel H., Martinez G., Cervellione K., Zafonte V.B., Gafoor K., Bagheri F. (2020). Marked Elevation in Serum Procalcitonin Levels Do Not Correlate with Severity of Disease or Mortality in Hospitalized Patients: A Retrospective Study. Biomark. Insights.

[37] Agnello L., Giglio R.V., Bivona G., Scazzone C., Gambino C.M., Iacona A., Ciaccio A.M., Lo Sasso B., Ciaccio M. (2021). The Value of a Complete Blood Count (CBC) for Sepsis Diagnosis and Prognosis. Diagnostics.

[38] Simon L., Gauvin F., Amre D.K., Saint-Louis P., Lacroix J. (2004). Serum procalcitonin and C-reactive protein levels as markers of bacterial infection: A systematic review and meta-analysis. Clin. Infect. Dis..

[39] An A.Y., Baghela A., Zhang P., Falsafi R., Lee A.H., Trahtemberg U., Baker A.J., Dos Santos C.C., Hancock R.E.W. (2023). Severe COVID-19 and non-COVID-19 severe sepsis converge transcriptionally after a week in the intensive care unit, indicating common disease mechanisms. Front. Immunol..

[40] Perschinka F., Mayerhofer T., Lehner G.F., Hasslacher J., Klein S.J., Joannidis M. (2022). Immunologic response in bacterial sepsis is different from that in COVID-19 sepsis. Infection.

[41] Yu J., Wang Y., Lin S., Jiang L., Sang L., Zheng X., Zhong M. (2021). Severe COVID-19 has a distinct phenotype from bacterial sepsis: A retrospective cohort study in deceased patients. Ann. Transl. Med..

[42] Vitner E.B., Achdout H., Avraham R., Politi B., Cherry L., Tamir H., Yahalom-Ronen Y., Paran N., Melamed S., Erez N. (2021). Glucosylceramide synthase inhibitors prevent replication of SARS-CoV-2 and influenza virus. J. Biol. Chem..

[43] Trovato F.M., Mujib S., Jerome E., Cavazza A., Morgan P., Smith J., Depante M.T., O’Reilly K., Luxton J., Mare T. (2022). Immunometabolic analysis shows a distinct cyto-metabotype in Covid-19 compared to sepsis from other causes. Heliyon.

[44] Woznica E.A., Inglot M., Woznica R.K., Lysenko L. (2018). Liver dysfunction in sepsis. Adv. Clin. Exp. Med..

[45] Grammatikos G., Ferreiros N., Waidmann O., Bon D., Schroeter S., Koch A., Herrmann E., Zeuzem S., Kronenberger B., Pfeilschifter J. (2015). Serum Sphingolipid Variations Associate with Hepatic Decompensation and Survival in Patients with Cirrhosis. PLoS ONE.

[46] Velazquez A.M., Roglans N., Bentanachs R., Gene M., Sala-Vila A., Lazaro I., Rodriguez-Morato J., Sanchez R.M., Laguna J.C., Alegret M. (2020). Effects of a Low Dose of Caffeine Alone or as Part of a Green Coffee Extract, in a Rat Dietary Model of Lean Non-Alcoholic Fatty Liver Disease without Inflammation. Nutrients.

[47] Seah J.Y.H., Chew W.S., Torta F., Khoo C.M., Wenk M.R., Herr D.R., Choi H., Tai E.S., van Dam R.M. (2020). Plasma sphingolipids and risk of cardiovascular diseases: A large-scale lipidomic analysis. Metabolomics.

[48] Hammad S.M., Pierce J.S., Soodavar F., Smith K.J., Al Gadban M.M., Rembiesa B., Klein R.L., Hannun Y.A., Bielawski J., Bielawska A. (2010). Blood sphingolipidomics in healthy humans: Impact of sample collection methodology. J. Lipid Res..

