# Supplementary material for: Hexosylceramide Species in the Blood Decline in Both COVID-19 and Non-COVID-19 Sepsis
Source: Biomedicines. 2026 Jul 20;14(7):1635. doi: 10.3390/biomedicines14071635 (PMC13406877; doi:10.3390/biomedicines14071635)
Supplement: Supplementary file 1 [file biomedicines-14-01635-s001.zip › biomedicines-4383542-supplementary.pdf]

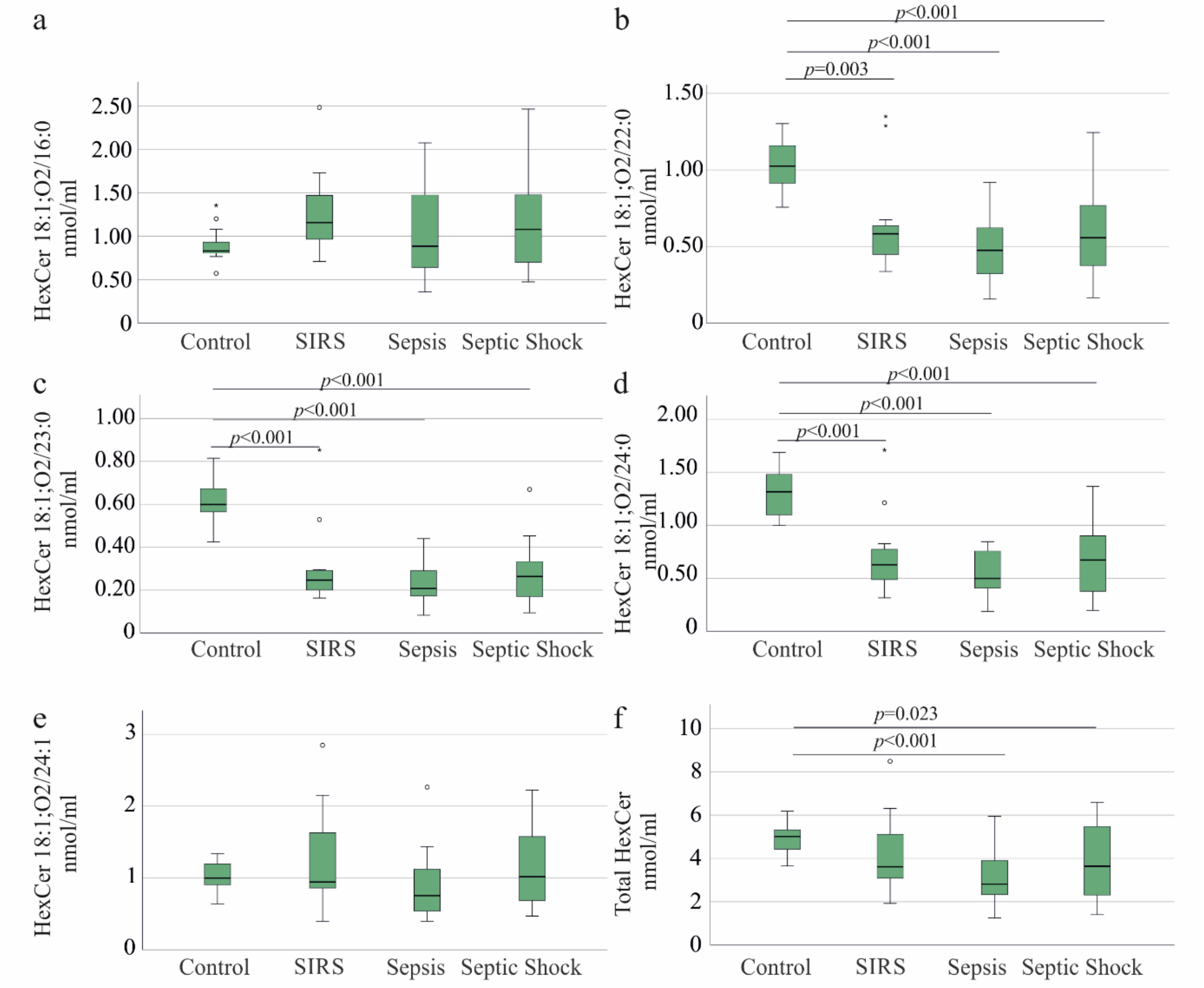

Figure S1. Hexosylceramide (HexCer) species in plasma of female controls, patients with systemic inflammatory response syndrome (SIRS), sepsis, or septic shock. (**a**) HexCer18:1;O2/16:0; (**b**) HexCer18:1;O2/22:0; (**c**) HexCer18:1;O2/23:0; (**d**) HexCer18:1;O2/24:0; (**e**) HexCer18:1;O2/24:1; (**f**) total HexCer levels in plasma of controls, patients with SIRS, sepsis, or septic shock. Outliers are represented by circles and small asterisks.

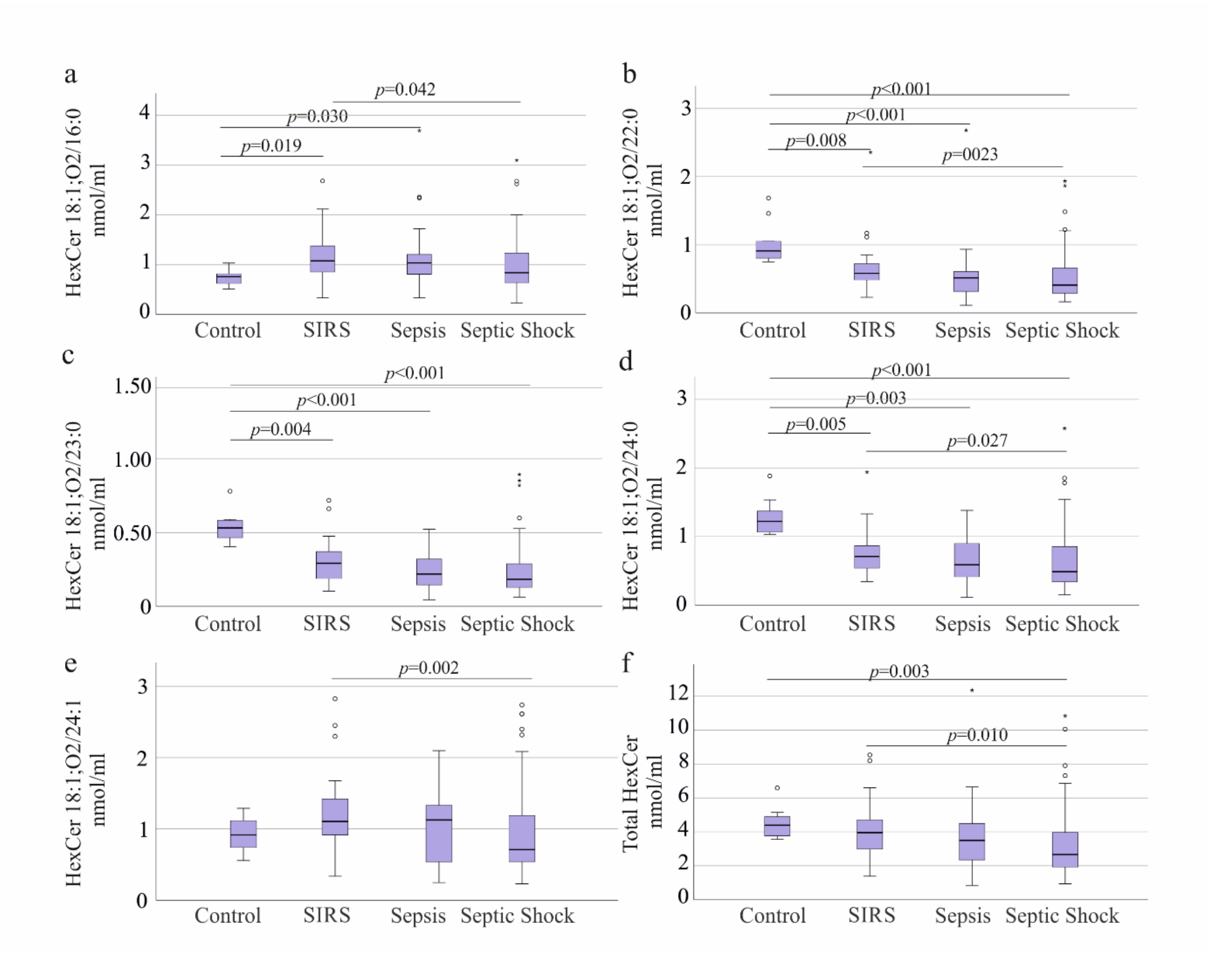

Figure S2. Hexosylceramide (HexCer) species in plasma of male controls, patients with systemic inflammatory response syndrome (SIRS), sepsis, or septic shock. (**a**) HexCer18:1;O2/16:0; (**b**) HexCer18:1;O2/22:0; (**c**) HexCer18:1;O2/23:0; (**d**) HexCer18:1;O2/24:0; (**e**) HexCer18:1;O2/24:1; (**f**) total HexCer levels in plasma of controls, patients with SIRS, sepsis, or septic shock. Outliers are represented by circles and small asterisks.

Table S1. Characteristics of patients with non-COVID-19 and COVID-19 sepsis. Numbers in superscript refer to patients for whom these data were available when data were not collected from the entire cohort. Data are presented as median (minimum–maximum). Statistical tests used: Kruskal-Wallis test and Chi-squared test.

**Parameters**	**Non-COVID-19**	**COVID-19**	**p-value**
Males/Females	95/40 (**n = 135)**	16/8 (**n = 24)**	
SIRS/Sepsis/Septic Shock	39/38/58	0/3/21	< 0.001
Age, years	58 (21–93)	63 (29–80)	
Body mass index, kg/m^2	26.2 (15.4–55.6) [p = 0.031]	28.3 (20.8–45.3)	
C-reactive protein, mg/L	161 (12–697)	124 (44–472)	
Procalcitonin, ng/mL	1.69 (0.05–270.00)	0.56 (0.08–65.40)	0.021
Leukocytes, n/nL	10.52 (0.06–1586.00)	8.74 (2.78–18.47)	
Neutrophils, n/nL	7.92 (0–70.20) [130]	6.59 (0–48.40) [23]	
Basophils, n/nL	0.04 (0–0.90) [131]	0.03 (0–0.60) [23]	
Eosinophils, n/nL	0.14 (0–2.89) [131]	0.02 (0–8.80) [23]	0.003
Monocytes, n/nL	0.79 (0–45.00) [131]	0.64 (0–10.90)[23]	
Lymphocytes, n/nL	0.96 (0.08 –16.80) [131]	0.69 (0.04 –10.90)[23]	
Immature granulocytes, n/nL	0.10 (0–6.19) [129][p < 0.001]	0.24 (0–3.84) [23]	
Total bilirubin, mg/dL	1.20 (0.10–36.60) [129]	0.65 (0.20–7.30) [22]	0.007
Albumin, g/L	21.9 (6.3–42.0) [126]	28.1 (20.2–36.6) [21]	0.001
Aspartate aminotransferase, U/L	44 (6–1597) [122]	54 (23–126) [22]	
Alanine aminotransferase, U/L	30 (6–770) [123]	36 (11–283) [19]	
Gamma-glutamyl transferase, U/L	115 (11–1093) [113]	198 (22–1266) [14]	
Total cholesterol nmol/ml	2229 (712–8529)	2804 (989–5727)	0.026
Vasopressor therapy	74	22	< 0.001
Dialysis	43	11	
Ventilation	73	24	< 0.001

Table S2. Characteristics of patients with non-COVID-19 and COVID-19 sepsis requiring ventilation. Numbers in superscript refer to patients for whom these data were available when data were not collected from the entire cohort. Data are presented as median (minimum–maximum). Statistical tests used: Kruskal-Wallis test and Chi-squared test.

**Parameters**	**Non-COVID-19**	**COVID-19**	**p-value**
Males/Females	52/21 (**n = 73)**	16/8 (**n = 24)**	
SIRS/Sepsis/Septic Shock	4/15/54	0/3/21	
Age, years	58 (21–93)	63 (29–80)	
Body mass index, kg/m^2	26.7 (15.4–53.6) [72]	28.3 (20.8–45.3)	
C-reactive protein, mg/L	170 (18–697)	124 (44–472)	
Procalcitonin, ng/mL	2.29 (0.10–114.40)	0.56 (0.08–65.40)	0.010
Leukocytes, n/nL	10.98 (0.32–1586.00)	8.74 (2.78–18.47)	
Neutrophils, n/nL	8.96 (0.01–70.20) [72]	6.59 (0–48.40) [23]	
Basophils, n/nL	0.04 (0.01–0.90) [72]	0.03 (0–0.60) [23]	
Eosinophils, n/nL	0.16 (0–1.75) [72]	0.02 (0–8.80) [23]	0.002
Monocytes, n/nL	0.84 (0.05–9.70) [72]	0.64 (0–10.90)[23]	
Lymphocytes, n/nL	1.10 (0.08 –16.80) [72]	0.69 (0.04 –10.90)[23]	
Immature granulocytes, n/nL	0.14 (0.00–6.19) [72]	0.24 (0–3.84) [23]	
Total bilirubin, mg/dL	1.60 (0.30–26.00) [69]	0.65 (0.20–7.30) [22]	0.002
Albumin, g/L	21.6 (6.30–42.0) [69]	28.1 (20.2–36.6) [21]	< 0.001
Aspartate aminotransferase, U/L	46 (8–1597) [66]	54 (23–126) [22]	
Alanine aminotransferase, U/L	27 (7–770) [67]	36 (11–283) [19]	
Gamma-glutamyl transferase, U/L	95 (11–799) [59]	198 (22–1266) [14]	
Total cholesterol nmol/ml	2097 (904–6840)	2805 (989–5727)	0.012
Vasopressor therapy	63	22	
Dialysis	40	11	
